# Immunoprofiling of Cell Wall Carbohydrate Modifications During Flooding-Induced Aerenchyma Formation in Fabaceae Roots

**DOI:** 10.3389/fpls.2019.01805

**Published:** 2020-02-03

**Authors:** Timothy Pegg, Richard R. Edelmann, Daniel K. Gladish

**Affiliations:** ^1^ Department of Biology, Miami University, Oxford, OH, United States; ^2^ Center for Advance Microscopy & Imaging, Miami University, Oxford, OH, United States

**Keywords:** aerenchyma, pectin, de-methyl-esterification, PCD, homogalacturonan, root, lysigenous, legume

## Abstract

Understanding plant adaptation mechanisms to prolonged water immersion provides options for genetic modification of existing crops to create cultivars more tolerant of periodic flooding. An important advancement in understanding flooding adaptation would be to elucidate mechanisms, such as aerenchyma air-space formation induced by hypoxic conditions, consistent with prolonged immersion. Lysigenous aerenchyma formation occurs through programmed cell death (PCD), which may entail the chemical modification of polysaccharides in root tissue cell walls. We investigated if a relationship exists between modification of pectic polysaccharides through de-methyl esterification (DME) and the formation of root aerenchyma in select Fabaceae species. To test this hypothesis, we first characterized the progression of aerenchyma formation within the vascular stele of three different legumes—*Pisum sativum*, *Cicer arietinum*, and *Phaseolus coccineus*—through traditional light microscopy histological staining and scanning electron microscopy. We assessed alterations in stele morphology, cavity dimensions, and cell wall chemistry. Then we conducted an immunolabeling protocol to detect specific degrees of DME among species during a 48-hour flooding time series. Additionally, we performed an enzymatic pretreatment to remove select cell wall polymers prior to immunolabeling for DME pectins. We were able to determine that all species possessed similar aerenchyma formation mechanisms that begin with degradation of root vascular stele metaxylem cells. Immunolabeling results demonstrated DME occurs prior to aerenchyma formation and prepares vascular tissues for the beginning of cavity formation in flooded roots. Furthermore, enzymatic pretreatment demonstrated that removal of cellulose and select hemicellulosic carbohydrates unmasks additional antigen binding sites for DME pectin antibodies. These results suggest that additional carbohydrate modification may be required to permit DME and subsequent enzyme activity to form aerenchyma. By providing a greater understanding of cell wall pectin remodeling among legume species, we encourage further investigation into the mechanism of carbohydrate modifications during aerenchyma formation and possible avenues for flood-tolerance improvement of legume crops.

## Introduction

Flooding is among the most common and costly natural disasters inflicted upon agricultural lands (Doocy et al., 2013). Between 2005 and 2015, global economic losses of over $19 billion were incurred due to destruction of crops and erosion of arable land from flooding (Conforti et al., 2018). Increased coastal flooding and changes of annual precipitation are predicted to cause significant economic losses within the next century (Hirabayashi et al., 2013). To aid in mitigating the future economic impact of flooding damage on plants, significant research has been conducted in the field of crop improvement with regards to understanding plant adaptations to water immersion (Grover et al., 2000; Evans, 2004; Bailey-Serres et al., 2012; Valliyodan et al., 2016; Mustroph, 2018).

One adaptive mechanism plants utilize against flooding is the creation of aerenchyma (Drew et al., 1980; Jackson and Armstrong, 1999). Aerenchyma tissues are characterized by the formation of large, air-filled channels or cavities in the stems, leaves or roots in plant cortical or vascular tissues (Yamauchi et al., 2013; Takahashi et al., 2016). These cavities allow plants to tolerate hypoxic conditions induced through prolonged water immersion by maintaining oxygen levels sufficient for cellular respiration and reducing the number of cells utilizing oxygen (Evans, 2004; Postma and Lynch, 2011; Yamauchi et al., 2013). Additionally, oxygen from aerenchyma diffuses through the plant apoplast into the surrounding soil, which increases soil oxygen content and protects tissues from infection by bacteria and fungi favored by anaerobic conditions (Jackson and Armstrong, 1999; Cronk and Fennessy, 2009; Takahashi et al., 2016).

Aerenchyma is often classified as either primary aerenchyma, forming within cortical tissues, or secondary aerenchyma, forming from cell divisions of meristematic phellogen layers (Shimamura et al., 2010). Primary aerenchyma can be either schizogenous, forming through separation of middle lamella between cells, or lysigenous, utilizing programmed cell death (PCD) of specific cells and tissues to form new cavities (Gunawardena et al., 2001a; Evans, 2004; Ishizaki, 2015). Lysigenous aerenchyma may also be formed in non-cortical tissues, such as the stele of legume roots such as *Pisum sativum* (pea) (Rost et al., 1991; Gladish and Niki, 2000; Sarkar and Gladish, 2012; Pegg et al., 2018) and *Phaseolus coccineus* (scarlet runner bean) roots under conditions of flooding stress (Takahashi et al., 2016).

Lysigenous aerenchyma formation is known to involve PCD that utilizes modification and subsequent deconstruction of plant cell walls to create aerenchyma cavities (Gunawardena et al., 2001a; Sarkar and Gladish, 2012). The plant cell wall itself is a dynamic structure consisting of interlinking matrices of xyloglucan and cellulose microfibrils inside a network of hydrated pectic polysaccharides (i.e. pectins) (Carpita, 1996). Modification of cell wall pectic polysaccharides is of significance in many plant physiological processes, such as fruit ripening (Hyodo et al., 2013; Paniagua et al., 2014), leaf abscission (Lashbrook and Cai, 2008), pollen tube growth (Bosch and Hepler, 2005) and lateral root emergence (Vilches-Barro and Maizel, 2015).

The process of de-methyl esterification (DME) modifies the pectin backbone structure (i.e. homogalacturonan) within plant cell walls by removing methyl ester groups from α-(1–4)-linked D-galacturonic acid chains. (Wolf et al., 2009; Daher and Braybrook, 2015). As a result, negatively charged carboxyl groups are created that participate in cross-linking reactions with calcium cations (**Supplemental Figure 1**). These cross-linking interactions form an “egg box” structure of paired homogalacturonan chains that allows susceptibility to hydrolytic enzymatic degradation of the pectin backbone from polygalacturonase (**Supplemental Figure 2**) and pectate lyase activity that destabilizes the cell wall matrix (Ochoa-Villarreal et al., 2012; Pérez-Pérez et al., 2019).

DME activity has been previously identified during cortical aerenchyma development in several crop species such as *Zea mays* (maize) (Gunawardena et al., 2001a), *Oryza sativa* (rice) (Qu et al., 2016) and *Saccharum* sp. (sugarcane) (Leite et al., 2017). Aerenchyma development is suspected to rely on DME to initiate degradation of the cell wall matrix by forming homogalacturonan residues susceptible to enzymatic hydrolytic cleavage (Gunawardena et al., 2001b; Pegg et al., 2018). However, an investigation into the chemical structure of the DME residues near aerenchyma cavities has been performed on relatively few plants species (Sarkar et al., 2008; Leite et al., 2017; Pegg et al., 2018).

In this project, we addressed the potential role of pectin modification during root aerenchyma formation in three members of the legume family (Fabaceae): *P. sativum*, *Cicer arietinum*, and *P. coccineus*. Our results indicated that pectin DME occurs in select cell regions prior to or during the formation of lysigenous aerenchyma in these legume species and that variation in the degree of pectin methyl-esterification (ME) is significant to cavity formation. Additionally, evidence exists for the removal of associated cell wall polymers such as cellulose and xylan as a potential requirement for DME activity to occur during aerenchyma formation.

## Materials and Methods

### Seedling Growth and Flooding Treatment

Seedlings were grown according to method of Gladish and Niki (2000). For each species 20 seeds (*P. sativum* and *C. arietinum*), or 10 seeds (*P. coccineus*), were sown, per beaker, into 2 l beakers filled with 1800 ml of sterile, super-coarse vermiculite (Perlite Vermiculite Packaging Industries, Inc., USA), moistened with 650 ml of deionized water, and covered with aluminum foil. Beakers were placed into 25°C growth chambers for 5 d in complete darkness to initiate root growth. Three replicates for each flooding treatment (12, 24, and 48 h water immersion) and each control (0, 12, 24, 48 h without flooding) were created using a separate 2 l beaker for each replicate.

To perform flooding treatments, three sets of beakers (12, 24, and 48 h water immersion) were removed from growth chambers, placed under a laminar flow hood, and filled with sterile deionized water to the surface level of the vermiculite substrate. An additional three sets of beakers (12, 24, and 48 h non-flooded) corresponding to the same timepoint as the flooding treatments were also removed from growth chambers but were not flooded to serve as control samples. Three non-flooded beakers representing the 0-hour timepoint were harvested at that time. Remaining beakers were returned to 25°C growth chambers and removed at either 12 h, 24, or 48 h after flooding to be harvested for sectioning.

### Sectioning, Fixation and Embedding

Five to ten root segments were harvested from each species per flooding treatment or non-flooding control. Segments were cut with carbon steel razor blades (Electron Microscopy Services, USA) from either 1.5–5 cm (*P. sativum* and *P. coccineus*) or 3–7 cm (*C. arietinum*) away from root tips. Segments were fixed in 1% paraformaldehyde and 2% glutaraldehyde solution in deionized water for 24 h at 5°C. Segments were then washed 3× with deionized water (15 min per wash), embedded in 3.5% agarose (Sigma-Aldrich, CAS 9012-36-6, USA) at 40°C, solidified, mounted on stubs of epoxy resin, and sectioned at 100 µm thickness on a Vibratome Series 1000 Sectioning System (Ted Pella, Inc., Redding, CA, USA). Sections from each root were stored separately in three separate pools (per treatment, per species) in 0.1M tris-buffered saline solution (pH 7.4) with 0.1% sodium azide at 5°C.

### Histological Staining and Area Measurement

Randomly selected root sections from each species pool were stained with 0.1% toluidine blue O stain (Electron Microscopy Sciences, RT26074-05, Hatfield, PA, USA) for 20 s, then washed three times with deionized water. Sections were placed in deionized water on standard 1 mm glass slides, flanked by two 22 × 22 mm, No. 1 coverslips serving as spacers, and covered with a 24 × 60 mm, No. 1.5 coverslip. A minimum of three sections (one section per individual root) from each species were observed per time point using bright field illumination on a Nikon Eclipse E200 upright binocular light microscope (Nikon, USA) with a 20× dry objective. Each section was photographed with a 12.2-megapixel CMOS digital camera (Samsung Galaxy S8 SM-G950U, Samsung, USA). Average area for aerenchyma cavities (n = 3) in each legume species was calculated for 12, 24 and 48 h flooding timepoints by measuring the 2D surface area of sections at each timepoint with ImageJ software (National Institutes of Health, USA). Data was plotted as a bar chart displaying average values with standard error bars using Microsoft Excel (Microsoft, USA).

### Scanning Electron Microscopy

Randomly selected root sections from each species pool were placed in 1% osmium tetroxide in deionized water for 24 h. Sections were washed 3× with deionized water (15 min per wash), following by an ethanol dehydration series. Samples in 100% ethanol were CO_2_ critical point drying, and then gold sputter-coated for 90 s to obtain a coating of 20 nm thickness. Samples were viewed on a Zeiss Supra 35 VP FEG SEM at 10 keV and 7.4 mm working distance.

### Immunolocalization

Ten randomly selected sections from each species pool, for control (five sections) and experimental treatments (five sections), were placed into sterile 24-well cell culture plates and blocked with 7% normal goat serum (Thermo Fisher Scientific, USA) for 24 h at 5°C. Samples were washed 3× (15 min per wash) with 10 mM Tris-buffered saline (pH 7.4) containing 0.1% TWEEN-20 (TBST) then incubated with 1/20 dilutions of LM19 (PlantProbes, University of Leeds, UK), JIM7 or JIM5 (CCRC, University of Georgia, USA) monoclonal antibodies for 24 h at 5°C (**Supplemental Table 1**). After incubation, samples were washed three times with TBST buffer and treated with 1/500 dilution of IgG goat anti-rat secondary antibody conjugated to Alexa Fluor™ 647 fluorescent dye (Thermo Fisher Scientific, USA) for 24 h at 5°C while wrapped with Parafilm M sealing film and covered in aluminum foil. Samples were washed a final time with three changes of TBST buffer and mounted in 100% glycerol (Sigma-Aldrich, CAS 56-81-5, USA) on standard 1 mm glass slides. Slides were covered with 24 x 60 mm, No.1 coverslips with two 22 × 22 mm, No. 1 coverslips applied underneath to serve as spacers. Samples were stored at 5°C in darkness when not in use.

### Enzyme Treatment

Randomly selected roots sections from each species pool for the 48-hour flooding treatment timepoint were incubated according to vendor instructions in the following enzyme solutions at 50°C for 2 h: 4% Cellulase, 1% xylanase, 3% pectinase, and 4% Viscoenzyme L (Sigma-Aldrich, USA) in 0.05 M citrate buffer (pH 5.0). Positive control treatment entailed incubation of samples in 0.1 M sodium carbonate (pH 11.4) at 50°C for 2 h to fully de-methyl-esterify homogalacturonan on exposed surfaces of the sample and ensure binding by LM19 antibody. Negative control treatment entailed incubation of samples in 0.05 M citrate buffer (pH 5.0) at 50°C for 2 h to replicate standard LM19 binding pattern observed without enzyme pretreatments. Samples were then washed three times with TBST buffer, treated with LM19 primary monoclonal antibody, and incubated with secondary antibody conjugated to Alexa Fluor^®^ 647 (Thermo Fisher Scientific, USA) prior to mounting in 100% glycerol on 1 mm glass slides covered with 24 × 60 mm No.1 coverslips.

### Fluorescence Microscopy

Autofluorescence and immunostained tissue sections were observed on an Olympus FV500 Laser Scanning Confocal system (Olympus Corporation, USA) using 20×/0.70 NA and 40×/0.75 NA dry objectives. Excitation of aldehyde-induced autofluorescence and Alexa Fluor^®^ 647 dye was achieved with 405 nm and 633 nm laser diodes, respectively. Images were recorded using a Photometric HQ cooled CCD camera.

## Results

### Distinct Morphological Characteristics Accompany Aerenchyma Development in Select Fabaceae Species

**Figure 1:**
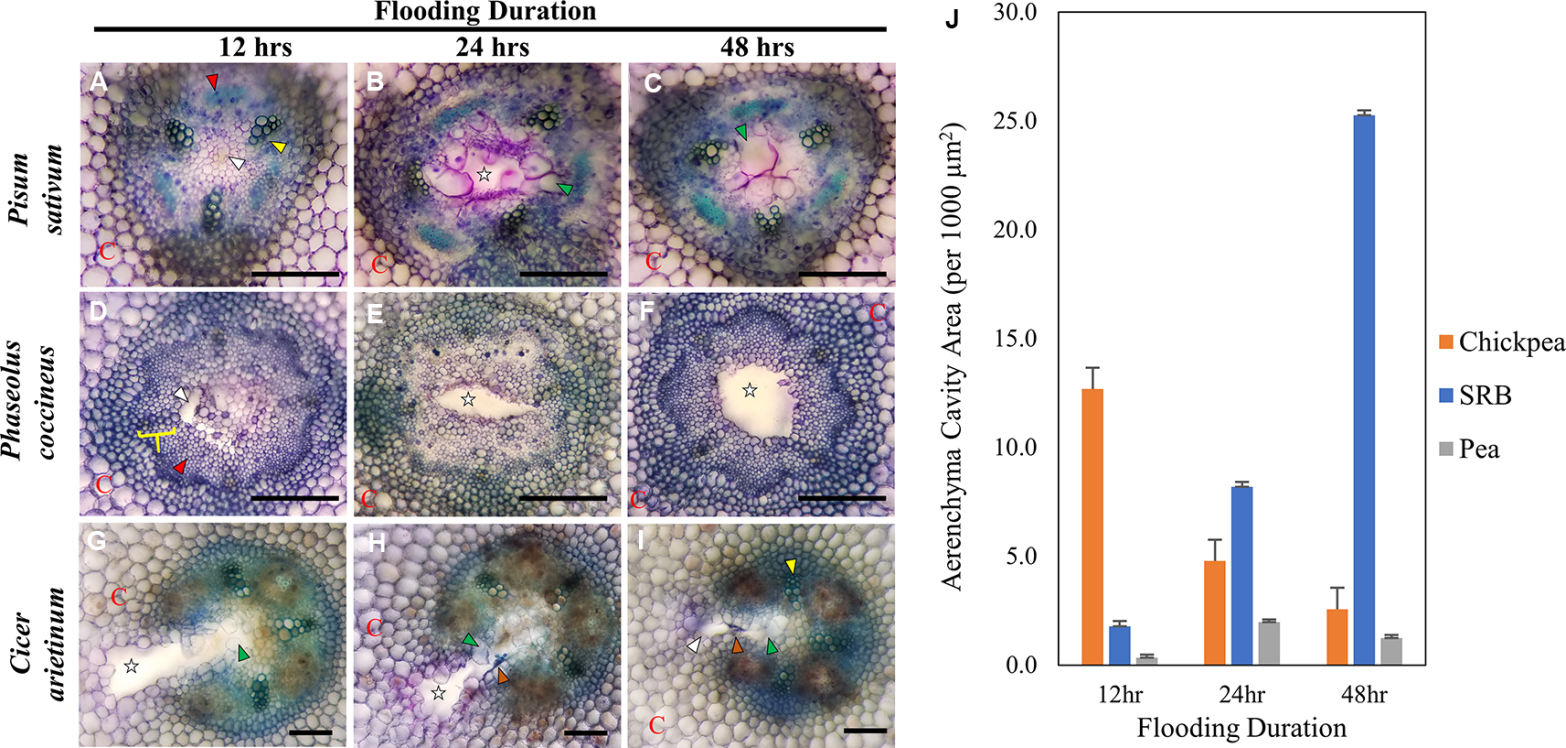
Toluidine Blue Staining of three Fabaceae root species during a 48-hour flooding time course: **(A**–**C)** *Pisum sativum* (pea), **(D**–**F)** *Phaseolus coccineus* (scarlet runner bean, SRB), **(G**–**I)** *Cicer arietinum* (chickpea). **(J)** Average area measurement of aerenchyma cavities across legume species and flooding timepoints with standard error bars (n = 3). Aerenchyma cavities indicted with white stars and wedges. Xylem and phloem indicated with yellow wedges/brackets and red wedges, respectively. C = cortex. Tylose-like cells (TLCs) indicated with green wedges. Degraded cell wall components (dark blue accumulations) indicated with orange arrows. Scale Bars = 100 µm.

**Figure 2:**
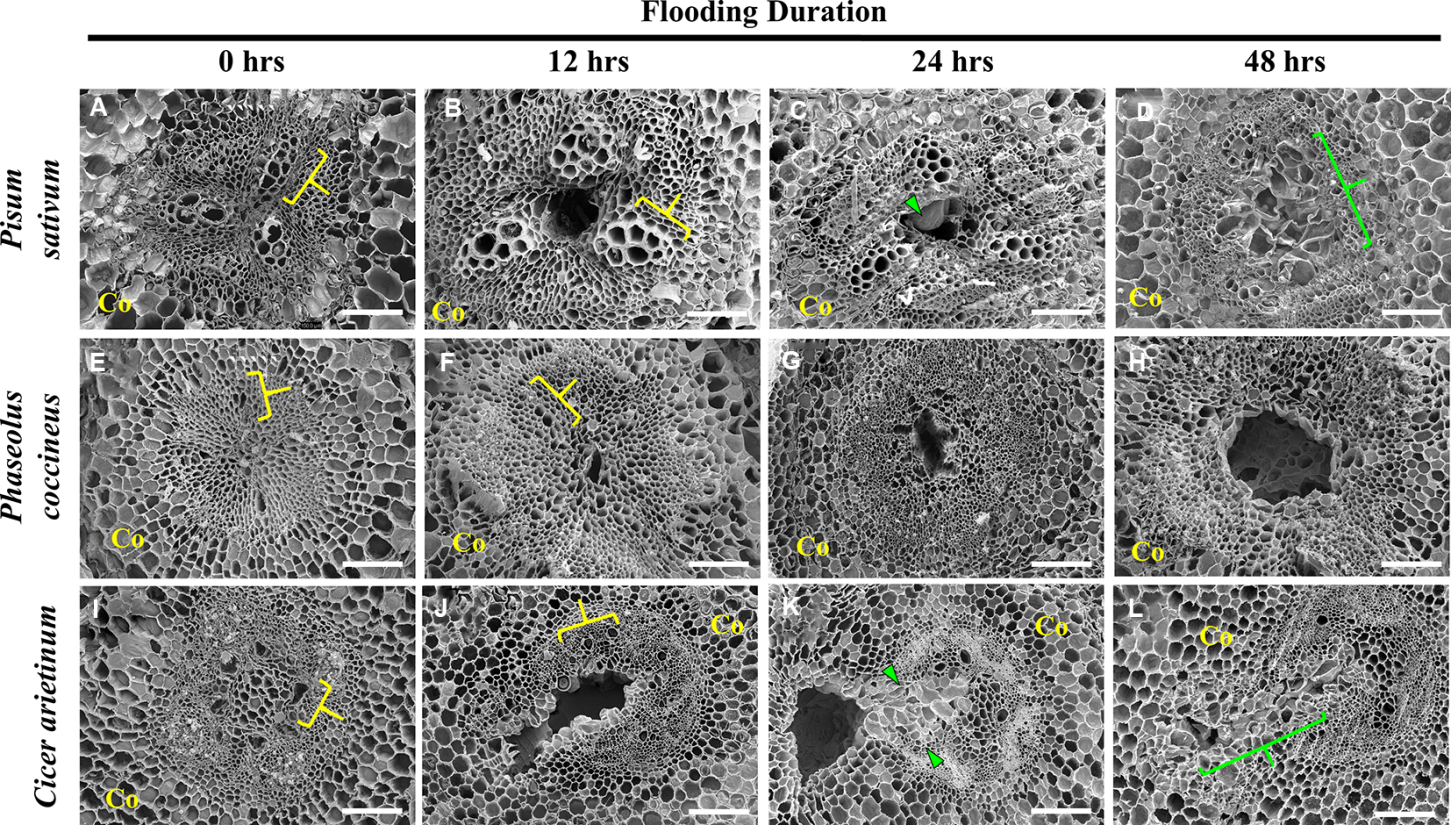
Scanning electron micrographs of aerenchyma formation in the Fabaceae species. **(A**–**D)** *Pisum sativum*, **(E**–**H)** *Phaseolus coccineus*, and **(I**–**L)** *Cicer arietinum* root cross sections displaying cavity formation in vascular tissue over a 48-hour flooding time course. Xylem indicated by yellow brackets. Tylose-Like Cells (TLCs) indicated with green wedges and brackets. Co = cortex. Scale bars = 100 µm.

In this study, we used histological staining and scanning electron microscopy (SEM) to examine the morphogenesis of aerenchymatous cavities in Fabaceae. Toluidine blue staining and SEM of three species, *P. sativum*, *C. arietinum* (chickpea), and *P. coccineus*, during a 48-hour flooding time course revealed similarities and differences in cell wall chemistry and morphological dimensions (**Figures 1** and **2**, **Supplemental Table 2**) experienced by the root vascular stele.

*P. sativum* aerenchyma formation was consistently observed at 12 h after flooding stress was induced (**Figures 1A** and **2B**). Cavity formation began near the metaxylem of one xylem pole within the stele and expanded to form a transversely circular aerenchymatous space that occupied the center of the stele (**Figure 2B**). Release of large bubbles during cross sectioning of *P. sativum* suggests these cavities were filled with air. Consistent with previous reports (Lu et al., 1991; Niki et al., 1998) aerenchyma became partly occluded with new tissue expanding from the margin of the vascular cavity within 24-48 h of flooding (**Figures 1B, C, J** and **2C, D**). We described these tissues as being composed of large, nucleated “bubble-like” cells that we name “tylose-like cells” (TLCs) due to their cosmetic resemblance to tyloses found in xylem vessels of various hardwoods (Esau, 1965; Carlquist, 2013; Leśniewska et al., 2017). Interestingly, toluidine blue stained tissue near the margins of the aerenchyma and the TLCs a bright magenta color that was not found elsewhere in the root cross section (**Figures 1B, C**).

*P. coccineus* aerenchyma formation followed a similar pattern as *P. sativum* with initiation adjacent to metaxylem (**Figures 1D** and **2F**) and creation of a transversely ovoid or circular cavity that occupied the center of the stele (**Figures 1F** and **2G, H**). Release of large bubbles from the aerenchyma during cross sectioning suggests these cavities were filled with air, similar to observations made in *P. sativum*. Unlike *Pisum*, *Phaseolus* aerenchyma formation did not entail creation of TLCs at any point within a 48-hour flooding treatment (**Figures 1F, J** and **2H**). Occasionally, *Phaseolus* sections showed large, circular remains of degraded cell tissue deep within aerenchyma (**Figure 1H**). Similar to *P. sativum*, application of toluidine blue resulted in cells bordering the aerenchyma staining a bright magenta color (**Figures 1D–F**).

**Figure 3:**
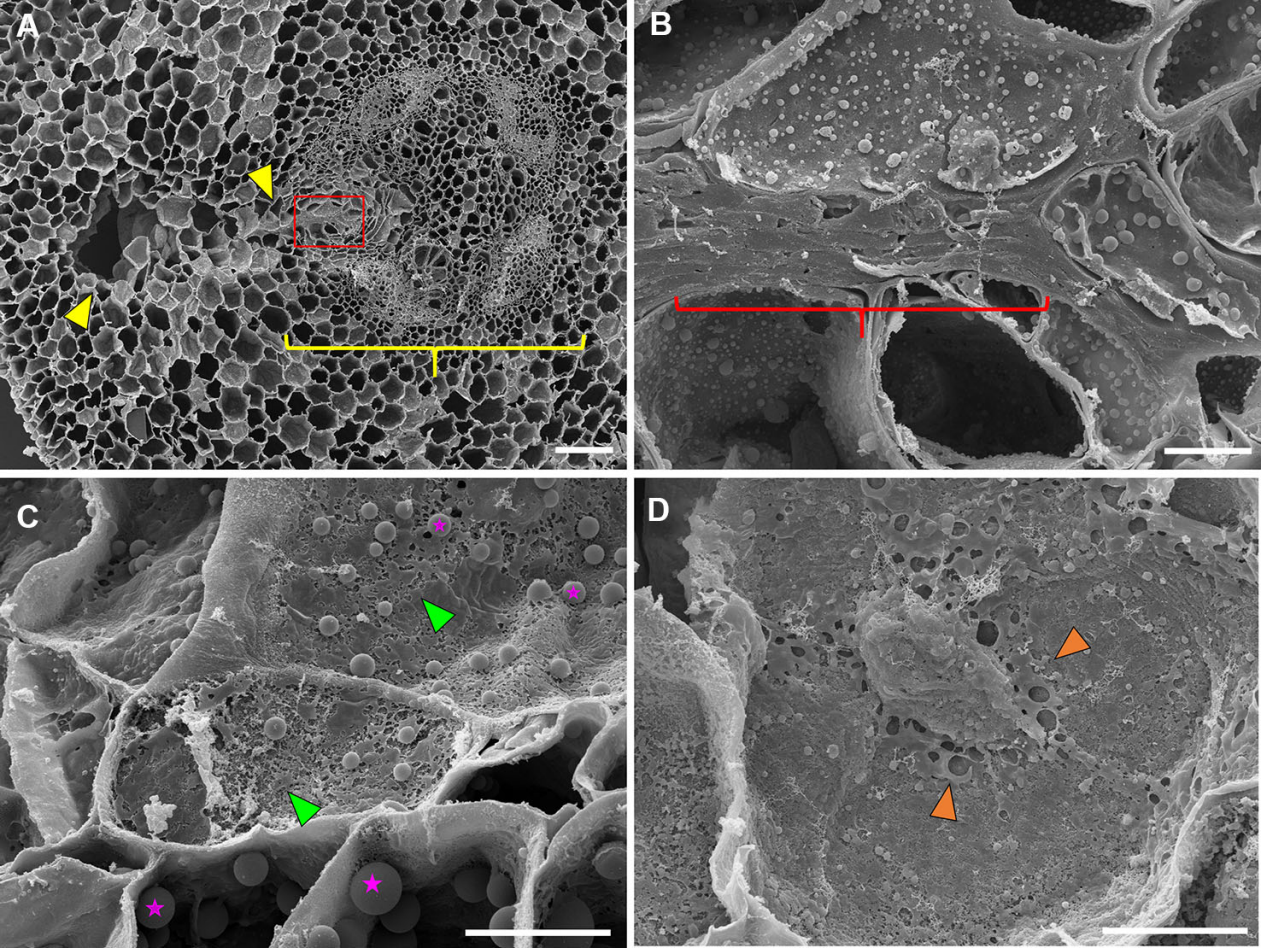
Scanning electron microscopy images of 48 hr-flooded *Cicer arietinum* (chickpea) root sections during aerenchyma formation. **(A)** Vascular stele (yellow bracket) with region of active cavity formation (yellow wedges), 100×. **(B)** Collapse and compression of cell walls near the edge of the vascular stele (yellow bracket) as seen in the magnified area highlighted in red from **(A)**, 1,500×. **(C)** Degradation of cell walls indicated by “pock-mocked” appearance (green wedges) and increased abundance of suspected storage plastids (magenta stars). **(D)** Accumulation of cell wall components in apoplast space (orange wedges) at 2,000× magnification. Scale bars at **(A)** 100 µm and **(B**–**D)** 10 µm.

**Figure 4:**
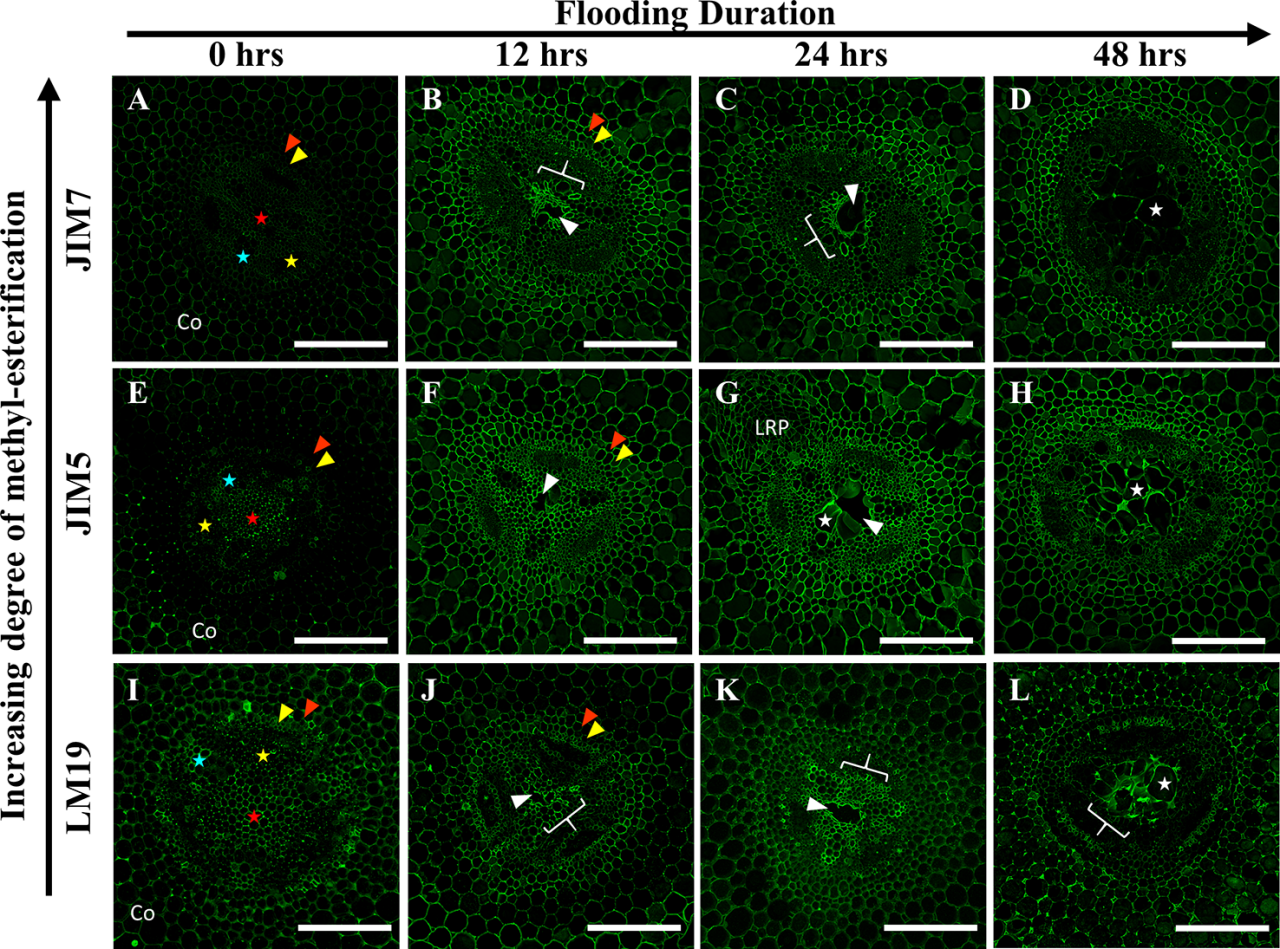
Localization patterns of ME and DME homogalacturonan in *Pisum sativum* during aerenchyma formation. Micrographs demonstrating monoclonal antibody labeling of **(A**–**D)** JIM7, **(E**–**H)** JIM5, and **(I**–**L)** LM19 on cortex, endodermis (red wedge), pericycle (yellow wedge), xylem (blue star), phloem sieve tube elements (yellow star), and pith (red star) of root cross sections. Areas with prominent antibody labeling indicated with white brackets. Aerenchyma cavities indicated with white edges. Tylose-Like Cells indicated with white stars. Co, cortex, LRP, lateral root primordia. Scale bars = 100 µm.

*C. arietinum* aerenchyma formation was quite distinct from either *P. sativum* or *P. coccineus*. Large, transversely oblong cavities were observed as early as 12 h after flooding (**Figure 1**, **Supplemental Table 2**), with a unidirectional expansion of aerenchyma over time, which began near the stele xylem poles and extended into the root cortex (**Figures 1G** and **2J**), though notable examples were observed of aerenchyma formation remaining confined within the stele (**Supplemental Figure 3**). Formation of a cavity appeared to separate and split portions of the xylem poles (**Supplemental Figures 4B–D**) that were previously intact (**Supplmental Figure 4A**). Closer examination of TLCs formed during periods of flooding stress revealed occasional accumulations of collapsed cells surrounded by TLC walls (**Figure 3B**) and characteristic signs of enzymatic activity, as indicated by “pooling” of degraded cellular components (**Figure 3D**). Degradation of these cells appeared to occur concurrently with TLC formation within the stele (**Figures 1H, I** and **2K, L**). Endodermis and pericycle layers appeared to be more resistant to degradation compared to other cortical and vascular tissues, which resulted in an “hourglass-shaped” aerenchyma cavity observed in some cross sections (**Figures 1H** and **2K, L**). Air most likely fills the aerenchyma due to bubble release during sectioning, similar to observations made earlier in the experiment for *P. sativum* and *P. coccineus*. By 48 h after initial exposure to flooding aerenchyma had been mostly filled with TLCs (**Figures 1I** and **2L**), resulting in severely diminished cavity size (**Figure 1J**), in a fashion similar to *P. sativum*. In addition, near the margins of aerenchyma within the cortex of *C. arietinum* roots toluidine blue stained cells a bright magenta (**Figures 1G–I**), similar to observations made in TLCs of *P. sativum* (**Figures 1B, C**) and borders of aerenchyma in *P. coccineus* (**Figures 1D–F**), which suggests a similar chemical modification has occurred in these cell walls.

### Immunolabeling of Fabaceae Root Radial Sections Indicates Specific Degrees of Pectin De-Methyl Esterification Adjacent to Aerenchyma

To evaluate the significance of cell wall pectin modification during aerenchyma formation, we labeled each Fabaceae species with three monoclonal antibodies targeting homogalacturonan pectin residues with differing degrees of de-methyl esterification (DME): LM19 (DME homogalacturonan), JIM5 (partially DME homogalacturonan), and JIM7 (fully methylated homogalacturonan). Immunolabeling of *P. sativum* flooding-time course series sections showed binding by LM19, JIM5 and JIM7 antibodies within central parenchyma, metaxylem, cortical apoplast and cells near phloem sieve tube elements (**Figure 4**). During aerenchyma formation, 12 and 24 h after flooding, binding by LM19 and JIM5 antibodies was detected within the cell walls and middle lamella of four to six cell layers adjacent to forming aerenchyma cavities (**Figures 4F, G, J, K**). Binding of JIM7 appeared to indicate a similar localization pattern within cells adjacent to aerenchyma but was more restricted and localized to three cell layers or less adjacent to the aerenchyma cavity (**Figures 4B, C**). All three antibodies labeled TLCs produced by roots flooded for 48 h, suggesting the presence of multiple DME and methyl esterified homogalacturonan epitopes (**Figures 4D, H, L**). Interestingly, the availability of the epitopes may be different based on chemical composition due to the observed “spottiness” of the JIM7 antibody binding pattern (**Figure 4D**) compared to JIM5 (**Figure 4H**) and JIM7 (**Figure 4L**).

**Figure 5:**
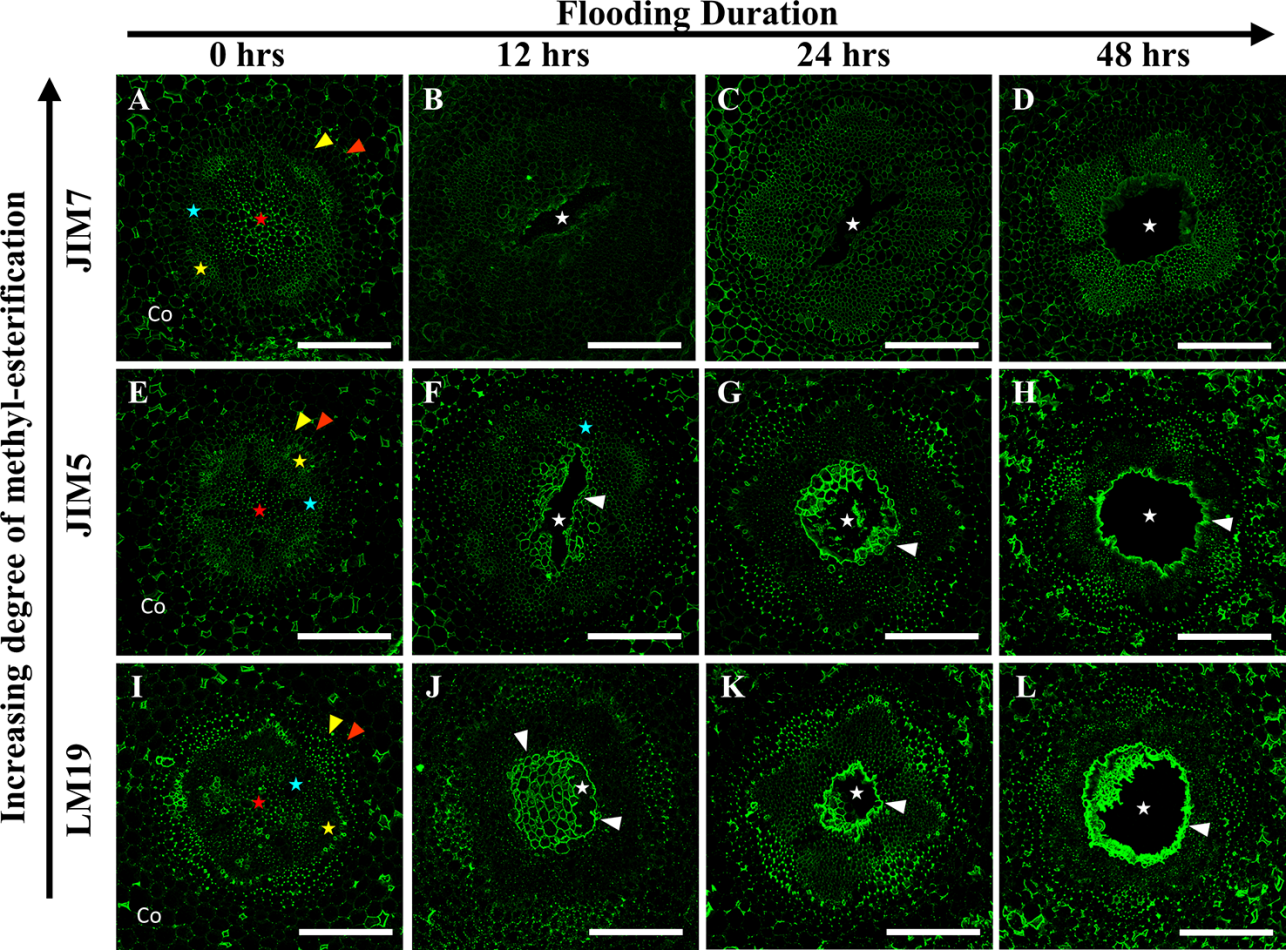
Localization patterns of ME and DME homogalacturonan in *Phaseolus coccineus* during aerenchyma formation. Micrographs demonstrating monoclonal antibody labeling of **(A**–**D)** JIM7, **(E**–**H)** JIM5, and **(I**–**L)** LM19 on cortex, endodermis (red wedge), pericycle (yellow edge), xylem (blue star), phloem sieve tube elements (yellow star), and pith (red star) of root cross sections. Cell layers prominently labeled with antibodies are indicated with white wedges. Aerenchyma cavities indicated with white stars. Co, cortex. Scale bars = 100 µm.

In *P. coccineus*, LM19, JIM5 and JIM7 antibodies displayed specific localization patterns within central parenchyma, cortical tissue apoplast, and cell walls of peripheral regions bordering the sieve tube elements (**Figures 5** and **7G–I**). By 12 h of flooding, all antibodies showed localization within cell walls and middle lamellas of central parenchyma cells within three to four cell layers of the aerenchyma, which suggests that de-methyl-esterification had probably begun. At 24–48 h after flooding, LM19 and JIM5 labeling was localized to most of the cell walls and middle lamellas of the root central parenchyma due to the increasing size of the aerenchyma cavity (**Figures 5G, H, K, L**). Similar to results seen in *P. sativum* (**Figure 4**), the binding pattern of JIM7 was noticeably less consistent and uniform compared to JIM5 and LM19 despite having shared localization patterns (**Figures 5A–D**).

**Figure 6:**
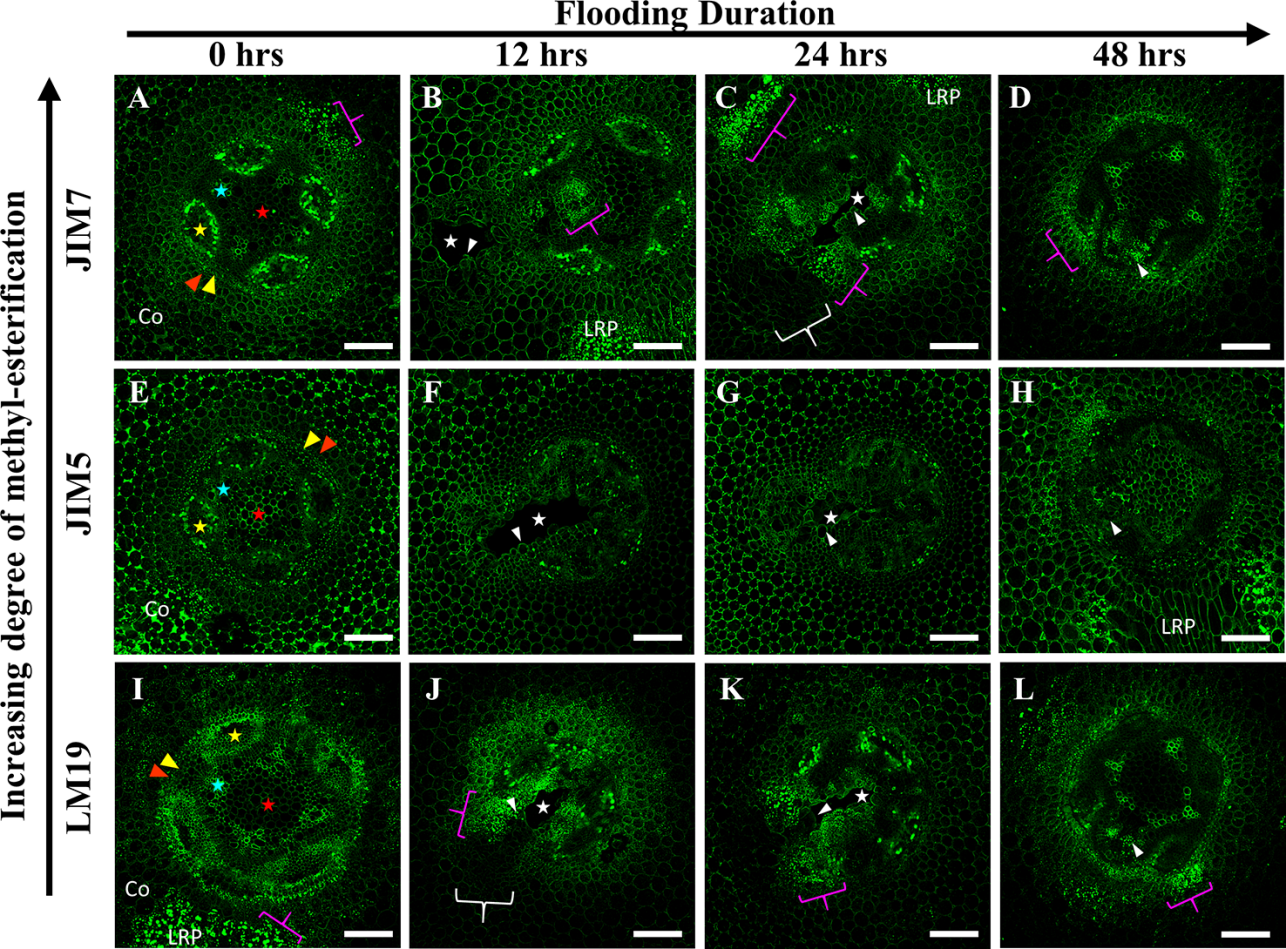
Localization patterns of ME and DME homogalacturonan in *Cicer arietinum* during aerenchyma formation. Micrographs demonstrating monoclonal antibody labeling of **(A**–**D)** JIM7, **(E**–**H)** JIM5, and **(I**–**L)** LM19 on cortex, endodermis (red wedge), pericycle (yellow edge), xylem (blue star), phloem sieve tube elements (yellow star), and pith (red star) of root cross sections. Speckling pattern (magenta brackets) indicate cells containing membrane-bound bodies (MBBs). Aerenchyma cavities indicated with white stars. Areas with poor or non-existent antibody labeling indicated with white brackets. Tylose-Like Cells (TLCs) indicated with white wedges. Co, cortex, LRP, lateral root primordia. Scale bars = 100 µm.

**Figure 7:**
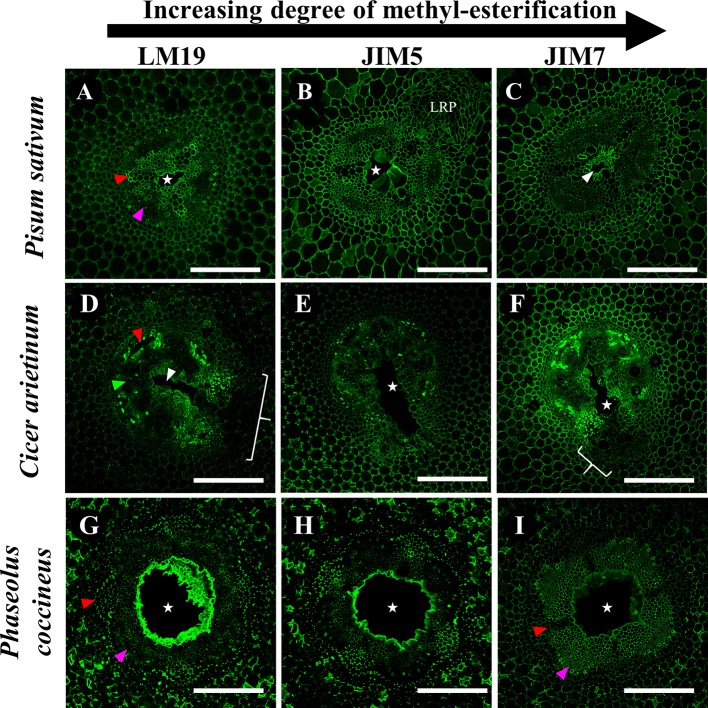
Immunolabeling of three Fabaceae species with monoclonal antibodies targeting pectin residues with varying degrees of de-methyl-esterification. **(A–C)** *Pisum sativum*, **(D**–**F)** *Cicer arietinum*, **(G**–**I)** *Phaseolus coccineus* root cross sections. Antibody labeling indicated with green false color. Areas with loss of antibody labeling signal indicated with white brackets. Micrographs contain labeled aerenchyma cavities (white stars and wedges), xylem (red wedges), and phloem (magenta wedges) in cross-sections from root segments. LRP, lateral root primordia. Scale bars = 100 µm.

Immunolabeling patterns for *C. arietinum* (**Figure 6**) were quite distinct from either *Pisum sativum* or *P. coccineus* (**Figures 4** and **5**). General localization patterns for LM19, JIM5 and JIM7 indicated the presence of all three antibody epitope structures in cortical apoplast, pericycle layer, xylem and cells bordering sieve tube elements of *Cicer* (**Figure 6**). Interestingly, LM19 and JIM7 antibody labeling was also prevalent on 0.5–1.0 µm membrane-bound bodies (MBB) found within cells of the pericycle, endodermis and inner cortical cell layers (**Figures 6A–D, I–L**), while it was mostly absent from similar tissues when labeled with JIM5 (**Figures 6E–H**). During aerenchyma formation, antibody labeling was limited to cell walls immediately adjacent to the cavity (**Supplemental Figures 3A–C**), newly formed TLCs (**Figures 6B–D, F–H, J–L**), or cell MBBs in the case of LM19 and JIM7 (**Figures 6B–D, J–L** and **7D, F**). Less consistent antibody binding patterns for LM19 and JIM7, compared to JIM5, was observed in cells adjacent to aerenchyma extending into the root cortex and TLCs developing within that region (**Figures 6B, C, J, K** and **7A, C**), which suggests an absence of fully DME and ME homogalacturonan. By comparison, JIM5 binding was prominent in cortical cells adjacent to aerenchyma, which implies the presence of partially DME homogalacturonan in these same cortical areas (**Figures 6F–H** and **7B**). However, JIM5 poor binding in the MBB of the inner cortex and stele, which contrasted with the consistent labeling observed from LM19 and JIM7 (**Figures 7D–F**).

### Enzyme Treatments Suggest Cell Wall Components Mask LM19 Epitope by Cell Wall Matrix

**Figure 8:**
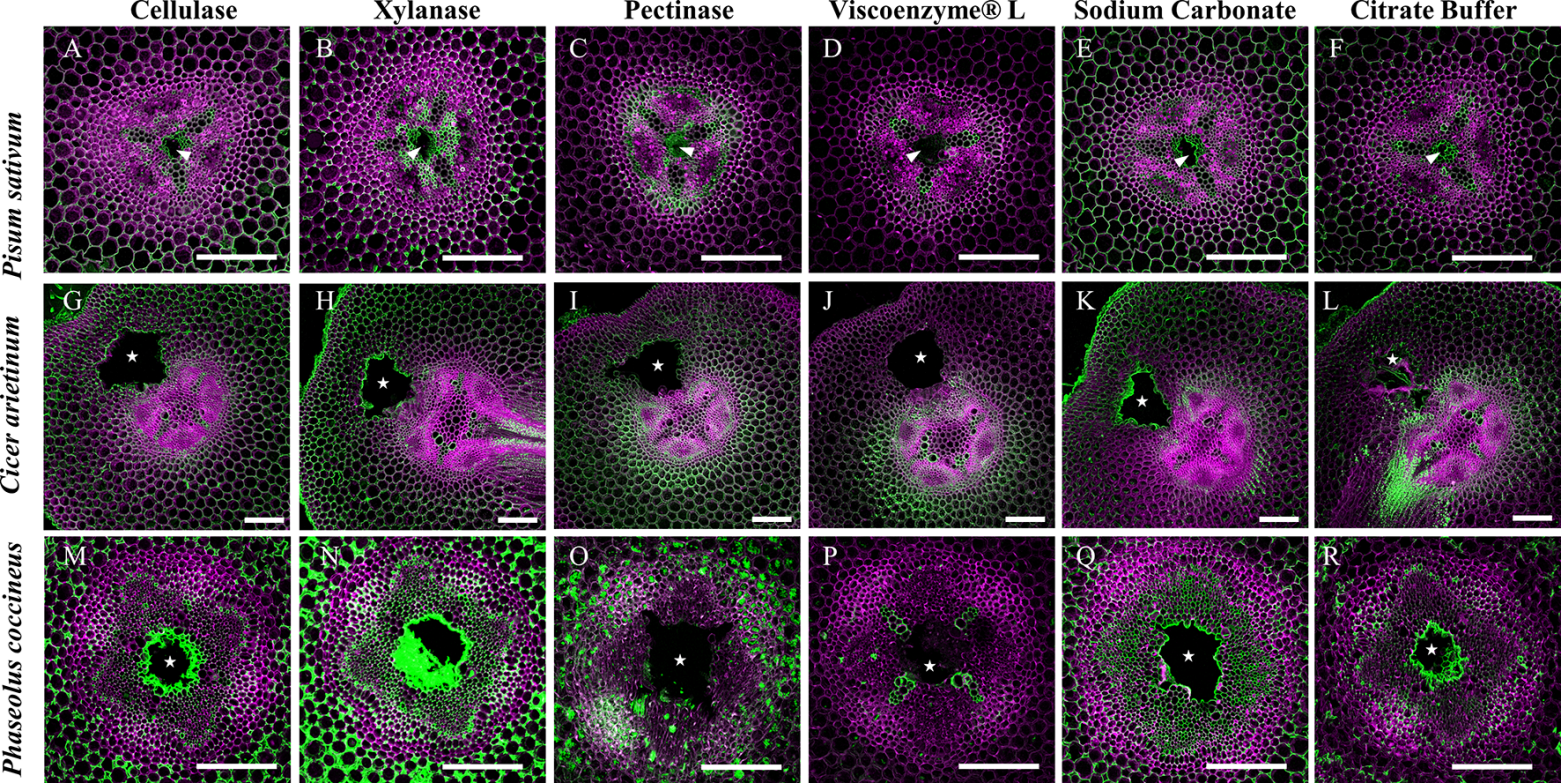
Effect of enzymatic pretreatments on LM19 labelling pattern in Fabaceae roots with aerenchyma. Fluorescent antibody labeling pattern of LM19 (green), composited with aldehyde induced fluorescence (magenta), to show distribution of de-methyl esterified homogalacturonan pectin in root cross sections. **(A, G, M)** Treatment of 2% cellulase, **(B**, **H**, **N)** 1% xylanase, **(C**, **I**, **O)** and 1% pectinase solutions. **(D**, **J**, **P)** Treatment of Viscoenzyme^®^ L enzyme solution (i.e. combination of cellulase, lichenase, pectinase, xylanase, etc.) to act as a negative control. **(E**, **K**, **Q)** Treatment of 0.1 M sodium carbonate, pH 11.4 solution to enhance LM19 binding pattern as a positive control. **(F**, **L**, **R)** Treatment of 0.05 M citrate buffer (negative control). White stars and wedges represent aerenchyma cavities. Scale bars = 100 µm.

Enzyme pretreatments of root sections before staining with LM19 antibody for DME homogalacturonan allowed evaluation of possible epitope site “masking” by other cell wall matrix components. Removal of cellulose prior to antibody labeling did not significantly alter LM19 localization pattern in either *P. sativum* or *C. arietinum* compared to sodium carbonate (**Figures 8A, D, E, H**) or citrate buffer control treatments (**Figures 8A, E–G, K, L**). However, cellulose removal in *P. coccineus* (**Figure 8M**) did increase LM19 localization pattern coverage in cell walls and middle lamella bordering the aerenchyma cavity and cortical apoplast when compared to sodium carbonate (**Figure 8Q**) and citrate buffer control treatments (**Figure 8R**). Xylan removal expanded LM19 binding pattern to cover the cortical apoplast in all species (**Figures 8B, H, N**) compared to control treatments (**Figures 8E, F, K, L, Q, R**) with visual changes in cortical apoplast binding consistency in *Pisum* and *Phaseolus*, and cell walls in tissue adjacent to aerenchyma in *Cicer*. Negative control treatments with pectinase (**Figures 8C, I, O**) and Viscoenzyme^®^ L enzyme cocktail (**Figures 8D, J, P**) resulted in removal of LM19 binding pattern for *Pisum* and *Phaseolus* (**Figures 8D, P**), but had little effect on *Cicer* outside of loss of antibody binding in cell walls of the outer cortical cell layers (**Figure 8J**). Interestingly, treatment with Viscoenzyme^®^ L enzyme cocktail altered the binding pattern of LM19 to permit labeling of xylem in *Phaseolus*, which suggests that removal of several cell wall polysaccharides is required for pectin in similarly lignified cell walls of this species to become available for antibody binding (**Figure 8P**).

## Discussion

The present study described shared characteristics, and notable differences among aerenchyma formation as a result of sudden flooding in three members of Fabaceae: *P. sativum*, *P. coccineus*, and *C. arietinum.* A unique characteristic of aerenchyma formation in Fabaceae is the location of the aerenchyma cavity within the root stele (Lu et al., 1991; Rost et al., 1991; Niki et al., 1995; Niki and Gladish, 2001). In all three species studied, aerenchyma formation was detected in stele tissues within 1.5–3 cm of the root apical meristem and became increasingly visible in older tissue zones away from the root tip. Similar to previous research (Gladish et al., 2006), we observed initiation of aerenchyma formation in the stele in central parenchyma cells adjacent to metaxylem followed by transverse expansion of the cavity to occupy most of the central parenchyma region in each species (**Figures 1** and **2**). However, in *C. arietinum* we observed a unique aerenchyma formation pattern characterized by cavity formation continuing into the inner cortex and resulting in a large, rectangular or hourglass-shaped cavity when viewed in cross section (**Figures 1G, H** and **2J, K**). The biological significance of the aerenchyma pattern in *C. arietinum*, and why it differs from that of *P. sativum* and *P. coccineus*, is unknown but it may influence survival time of *C. arietinum* in hypoxic conditions by reducing the number of extraneous, oxygen-consuming cells in roots, as has been noted in other work in *Z. mays* and *O. sativa* (Drew et al., 2000; Evans, 2004). Furthermore, extending the aerenchyma cavity into the root cortex may increase the volume of air that *C. arietinum* can conduct during hypoxic conditions as compared to *P. sativum* and *P. coccineus*. Aerenchyma of all three species contain air, as indicated by the release of bubbles during cross-sectioning of root tissues, along with confirmation of oxygen content in *P. coccineus* aerenchyma in intact roots by previous research (Takahashi et al., 2016). This suggests the possibility that increases in aerenchyma air volume, due to changes in aerenchyma cavity dimensions, may enable prolonged functioning of aerobic metabolic processes in root tissues exposed to low-oxygen conditions.

Our study also described the formation of previously reported space-filling parenchyma cells (Lu et al., 1991; Niki et al., 1998; Pegg et al., 2018) during periods of prolonged flooding stress that cosmetically resemble tyloses found in hardwood plants (Esau, 1965; Carlquist, 2013; Leśniewska et al., 2017). The biological significance of these “tylose-like cells” (TLCs) forming in *Pisum* and *Cicer* samples is unclear with respect to formation and eventual filling of aerenchyma cavities during periods of flooding stress. Tyloses are often observed within older xylem tissues of vascular plants as ingrowths of parenchyma cells that prevent or limit water transport as a response to drought stress or pathogenic infection (Pallardy, 2008; Zhao et al., 2014; Micco et al., 2016). In some species of Fabaceae, TLCs may serve a similar purpose by removing the airspace within the stele through replacement with parenchyma and repairing a structural weakness induce by prolonged presence of aerenchyma cavities (citation).

Further observation of legume root sections with scanning electron microscopy revealed characteristic signs of cell wall collapse and enzymatic degradation in cells adjacent to expanding cavities (**Figure 3**). This supports previous research which proposed that root aerenchyma formation in certain members of Fabaceae (Lu et al., 1991; Rost et al., 1991; Niki et al., 1995; Niki and Gladish, 2001), rice (Joshi and Kumar, 2012) and tomato (Kawase, 1981) is lysigenous in nature. Furthermore, our study noted that cell wall degradation was very localized within one to three cell layers of forming aerenchyma (**Figure 3**), and suggests that a carefully regulated and localized PCD mechanism is required to form aerenchyma in this plant family (Sarkar et al., 2008b; Sarkar and Gladish, 2012) while preventing an uncontrolled enlargement that would consume essential xylem and phloem vasculature within the root stele (Sarkar et al., 2008b; Sarkar and Gladish, 2012). This may be particularly important in the case of *C. arietinum* since the expanding cavity partially removes one of xylem poles in the tetrarch stele, which could require the conservation of the remaining three xylem bundles to ensure proper water conduction through the root. Development of lysigenous aerenchyma may also prevent inhibition of aerobic cellular respiration in legume roots by creating an internal oxygen-containing channel when the rhizosphere environment becomes hypoxic due to flooding (Drew et al., 2000; Evans, 2004; Takahashi et al., 2016).

Our research also revealed the presence of de-methyl-esterified (DME), partially DME and fully methyl-esterified (ME) pectin residues in the cell walls and middle lamella of stele and cortical tissues involved in aerenchyma formation. Previous research proposed that removal of methyl ester substitutions from the homogalacturonan domains of pectin enables degradation of cell walls by unblocking cleavage sites between pectin residues, which are then targeted by polygalacturonases, pectin lyases and similar hydrolytic enzymes (Dheilly et al., 2016). Our observations showed that DME pectin is spatially localized within one to three cell layers around aerenchyma and increases from partial and fully DME during aerenchyma development (**Figures 4–7**). This indicates a direct correlation between DME pectin formation and degradation of root cell walls. Furthermore, DME process may also strengthen the plant primary cell wall pectin matrix through interactions with calcium cations (Hocq et al., 2016), benefitting roots by increasing cell wall mechanical strength (Celus et al., 2018) near the forming aerenchyma to compensate for the structural weakness caused by a large air channel within the stele. This enhancement of cell wall strength would be particularly advantageous for plants such as *C. arietinum* which have non-symmetrical aerenchyma extending into cortex tissues with thin primary cell walls.

Notable changes in pectin methyl-esterification were also noted in TLCs in *P. sativum* and *C. arietinum.* Similar to cells and middle lamella destined for degradation, the TLCs were thoroughly labeled with antibodies against DME (LM19) and partially DME (JIM5) homogalacturonan (**Figures 4** and **6**). The presence of DME homogalacturonan in TLCs primary walls has not been previously described and is likely due to action of pectin methylesterase activity upon the homogalacturonan backbone. Removal of methyl esters from homogalacturonan promotes hydrolytic enzyme activity required for “loosening” of primary cell walls prior to wall expansion (Foster, 1967; Micco et al., 2016; Wu et al., 2018) and is a possible prerequisite for expansion of TLCs into the aerenchyma cavity. Additionally, de-methyl-esterification of pectin may permit enlargement of TLCs prior to development of secondary wall patterning (Goulao et al., 2011), as suggested by cell wall morphology observed in the present study (**Supplemental Figure 5**)

Enzyme treatments performed in this study indicated that hemicelluloses such as xylan, along with cellulose, may “mask” pectin from recognition by monoclonal antibodies targeting de-methyl esterified homogalacturonan residues (**Figure 8**, **Supplemental Figure 6**). Previous research suggests that “masking” occurs due to pectin and xylan binding to each other within primary cell wall matrices and physically blocking access of antibodies to epitope binding sites (Marcus et al., 2008). The presence of masking effects in our root samples suggests the possibility that pectin de-methyl-esterification may occur in a wider region of the central parenchyma than previously observed in the present study. This prediction was supported by results in *P. sativum* and *P. coccineus* (**Figures 8B, F, N, R**) where removal of xylan expanded LM19 antibody labeling into stele tissue further away from aerenchyma cavities. Non-flooded control samples also manifested this labeling pattern, but to a less consistent degree, suggesting that the flooding treatment itself may alter effectiveness of xylanase enzymatic pretreatment (**Supplemental** **Figures 6B, F, N, R**). Interestingly, antibody binding patterns for DME pectin do not appear to change noticeably following removal of either cellulose or xylan in *C. arietinum* root sections when compared to non-enzyme-treated controls in either flooded samples (**Figures 8G, H, L**) or non-flooded samples (**Supplemental Figures 6G, H, L**). These observations suggest that primary cell wall polysaccharides in *C. arietinum* may be organized differently compared to *P. sativum* and *P. coccineus*, thereby preventing or limiting masking effects on pectin residues. Additionally, small differences in LM19 binding pattern contiguity between flooded (**Figure 8**) and non-flooded (**Supplemental Figure 6**) samples indicate that immersion in water may subtly alter cell wall chemistry throughout root segments from these legumes. One possible consequence is an increase in the hydration of the primary cell wall, resulting in changes to molecular rigidity of the pectin cross-linking network (Vicré et al., 1999; Bidhendi and Geitmann, 2015; Lampugnani et al., 2018) and potential alteration of enzyme penetration and activity that would explain the differences observed in the pre-treatment protocol (**Figure 8**, **Supplemental Figure 6**).

Our research also suggests the possibility of stele regions with strong antibody labeling having masking effects negated by previous removal of other cell wall components. Removal of xylan, and to a lesser extent cellulose, in areas adjacent to forming aerenchyma appears to eliminate masking and create the DME pectin antibody binding patterns seen in this study (**Figures 8F, L, R**). This hypothesis is supported in our study by observations that LM19 binding patterns in root cross-sections treated with cellulase, xylanase, and sodium carbonate (**Figures 8A, B, E, G, H, K, M, N, Q**) are greatly expanded throughout root stele compared to control treatments (**Figures 8F, L, R**) with DME pectin localized near the aerenchyma cavity. As a result of our observations, we propose that aerenchyma formation may depend on activity of multiple cell wall remodeling enzymes (i.e. cellulase, xylanase) working together to achieve cell wall degradation. Specifically, xylanases and cellulases may degrade xylan and cellulose polysaccharides in advance of de-methyl-esterification of pectin by PME enzymes and subsequent degradation by pectinases.

Our findings in the present study provide directions for future research into regulation and localization of components essential to DME during aerenchyma formation. For example, we observed that fragments of degraded root stele tissue may be found inside aerenchyma during cavity formation (**Supplemental Figure 7**), yet the destination of pectins from degraded cells is unclear. In the case of *C. arietinum*, degraded pectin residues with specific degrees of DME may accumulate within MBB and be utilized to construct TLCs during later stages of flooding. Pectin residues may also enter the apoplast (de Freitas et al., 2012; Anderson, 2016) and may become incorporated into the primary walls and middle lamella of cells adjacent to forming aerenchyma cavities. Observed differential metachromatic staining of toluidine blue near aerenchyma cavities (**Figure 1**) could be the result of pH changes (O'Brien et al., 1964; Niki et al., 2014; Bergholt et al., 2018) in the apoplast and indicate liberated, negatively charged DME pectin residues forming during cell wall or middle lamella degradation (Yamada et al., 2015; Printz et al., 2016).

Additionally, the localization of calcium and cell wall remodeling enzymes (i.e. pectin methylesterase and pectin lyase) within legume stele tissues during aerenchyma formation requires elucidation. Calcium is mainly localized in the cell walls of plant tissues, accounting for 60–75% total calcium content (Demarty et al., 1984), though it is also present in the surrounding, fluid-filled apoplast (de Freitas et al., 2012). High localization of calcium ions could indicate susceptibility to enzyme degradation of cell walls by virtue of Ca^2+^ linkages between DME homogalacturonan residues (Grant et al., 1973; Wolf et al., 2009) and by serving as a signaling molecule in proposed ethylene signal transduction pathways that initiate PCD in cells adjacent to forming aerenchyma (He et al., 1996; Gunawardena et al., 2001b). Similarly, confirmation of elevated pectin methylesterase in cells fated to be degraded was found to correlate with high calcium concentrations: this provides supporting evidence in legume roots for extensive pectin DME during aerenchyma expansion (Goulao et al., 2007; Rajhi et al., 2011; de Freitas et al., 2012)

The regulation of gene expression leading to pectin DME during Fabaceae aerenchyma formation also remains unclear. Previous work in plants such as *Arabidopsis thaliana* (Mühlenbock et al., 2007) and *O. sativa* (Yamauchi et al., 2017) suggests the involvement of hydrogen peroxide (H_2_O_2_) in the formation of cortical lysigenous aerenchyma. Additional research involving *Z. mays* (Drew et al., 1980), *P. sativum* (Gladish and Niki, 2008) and *O. sativa* (Yamauchi et al., 2017) suggests ethylene signaling pathways may also play a role in cortical aerenchyma formation. These pathways are initiated by exposure to hypoxic, waterlogged conditions and result in gene expression for cell wall remodeling enzymes such as cellulases, xylanases, and pectinases (i.e. polygalacturonase and pectin lyase)(Waldenmaier, 2011) through transcription factors such as RAVs (Related-to-ABI3/VP1) identified in sugarcane (Li et al., 2011; Tavares et al., 2019). Involvement of downstream components for these pathways is supported in the results of the present study (**Figure 8**), which indicate cellulase and xylanase activity may degrade cell wall polymers (i.e. cellulose and hemicellulose, respectively) that partially “mask” (protect) pectin from enzymatic activities such as de-methyl-esterification and hydrolytic cleavage of homogalacturonan by pectinases (Voragen et al., 2009; Xue et al., 2013).

Research into genes involved in alternative processes, such as lateral root emergence, may identify similar functions during aerenchyma formation due to both events requiring cell wall remodeling to accommodate new structures within root tissues (Péret et al., 2009; Ishizaki, 2015; Porco et al., 2016; Leite et al., 2017). Specifically, genes involved in the auxin signaling pathway and cell wall remodeling genes such as those for auxin response factors in *A. thaliana* (Sénéchal et al., 2014) and polygalacturonases (PGLR, PGAZAT) in *O. sativa* (Kumpf et al., 2013) may have orthologs in legumes that also regulate pectin modification during aerenchyma formation. The potential presence of conserved cell wall remodeling genes among disparate plant families encourages research into controlled induction of aerenchyma *via* manipulation of an existing genetic framework for pectin modification and subsequent cell wall degradation in root tissues. Benefits of such work could lead to crop improvement with respect to increased tolerance to flooding, and, potentially, drought by plant root systems (Zhu et al., 2010; Nord et al., 2013).

## Conclusion

Initiation of aerenchyma formation in three Fabaceae species begins with degradation of root parenchyma cells near metaxylem of the stele. Expansion of aerenchyma cavities continues within the stele (*P. sativum* and *P. coccineus*) or from the stele and into cortical tissues (*C. arietinum*) unless halted by formation of tylose-like cells (TLCs) that fill aerenchyma of species such as *P. sativum* and *C. arietinum*. Modification of the pectin homogalacturonan backbone structure through de-methyl-esterification appears to be one mechanism by which cell walls and middle lamella of tissues in forming lysigenous aerenchyma are prepared for enzymatic degradation to permit PCD and enable cavity formation. Additionally, presence of fully and partially de-methyl-esterified homogalacturonan residues in cell walls of forming TLCs suggests these pectin structures are essential to development of TLCs that occlude aerenchyma of *P. sativum* and *C. arietinum*. Evidence exists for removal of cellulose and hemicellulose (xylan) in the cell walls of tissues adjacent to forming aerenchyma. Removal may occur prior to aerenchyma formation to allow de-methyl-esterification and/or enzyme access to pectin backbone structure.

## Data Availability Statement

The datasets generated for this study are available on request toeither the corresponding author, DG (gladisdk@miamioh.edu) or TP (peggtj@miamioh.edu).

## Author Contributions

TP performed the primary experimental work. TP performed seedling cultivation, tissue sectioning, absorbance dye staining, immunolabeling, enzyme treatments and image processing. RE provided technical assistance in microscope operation and protocol development. RE and DG evaluated experimental design, project progress and provided editing of manuscript. All authors performed data analysis, interpretation, and approved the final manuscript.

## Conflict of Interest

The authors declare that the research was conducted in the absence of any commercial or financial relationships that could be construed as a potential conflict of interest.
